# The ‘Comparative Logic’ and Why We Need to Explain Interlanguage Grammars

**DOI:** 10.3389/fpsyg.2021.717635

**Published:** 2021-10-25

**Authors:** Laura Domínguez, María J. Arche

**Affiliations:** ^1^Modern Languages and Linguistics, University of Southampton, Southampton, United Kingdom; ^2^School of Humanities and Social Sciences, Centre for Research and Enterprise in Language, University of Greenwich, London, United Kingdom

**Keywords:** comparative fallacy, native speaker, interlanguage, control group, Universal Grammar

## Abstract

In this paper we argue that Bley-Vroman’s Comparative Fallacy, which warns against comparisons between native speakers and learners in second-language acquisition (SLA) research, is not justified on either theoretical or methodological grounds and should be abandoned as it contravenes the explanatory nature of SLA research. We argue that for SLA to be able to provide meaningful explanations, grammatical comparisons with a baseline (usually of native speakers although not always the case) are not only justified but necessary, a position which we call the ‘Comparative Logic’. The methodological choices assumed by this position ensure that interlanguage grammars are analysed in their own right and respecting their own principles. Related issues, such as why we focus on the native speaker and why investigating deficits in linguistic-cognitive SLA is essential in our field are discussed as well.

## The Nature of Comparisons in Second-Language Acquisition Research

The view that comparisons between native speakers (NS) and non-native speakers (NNS), which are pervasive in second-language acquisition (SLA) research, should be discouraged is not new (Firth and Wagner, 1997; Klein, 1998). Recently, however, concerns about the use of these comparisons have been raised among some researchers working within the so-called linguistic-cognitive approaches to SLA^1^ arguing that comparing learners with natives falls into a ‘comparative fallacy’ (*CF*) as described by Bley-Vroman (1983) and help promote a monolingual bias in our field. The *CF* rests on two key claims: (1) the linguistic system of the learner [the interlanguage grammar (ILG)] is a system on its own right and (2) comparisons between ILG and other systems (including the target grammar) are not legitimate under any circumstances (see also Lakshmanan and Selinker, 2001). We argue, however, that these two claims are independent from each other. Indeed, many SLA researchers have explicitly claimed that the language of the second-language acquirers ‘represents a linguistic system in its own right and should be investigated as such (Huebner, 1983, p. 33)’; this view is consistent with Selinker’s (1972) original conception of ‘interlanguage’, and it is widely accepted in our field. The main concern for researchers (from all fields) is the legitimacy of NS-NNS comparisons (Firth and Wagner, 1997; Klein, 1998; Cook, 1999).^2^

According to the *CF*, ILG should be investigated without being compared with NS (the control group) as this may result in a view of learner grammars as ‘degenerate’ systems of less quality (i.e., the comparison necessarily presupposes a prejudice against NNS). In this paper, we position ourselves against this view (see also Mack, 1997; Montrul, 2013) and argue that, despite its increasing popularity among linguistic-cognitive SLA researchers, the *CF* is not justified on either theoretical or methodological grounds. Furthermore, we propose replacing the *CF* with the Comparative Logic which justifies comparisons with a baseline as these not only allow researchers to study L2 grammars ‘in their own right’ but are also essential in order to examine and explain the nature of L2 grammars.

In this paper, we will present and defend the Comparative Logic on the basis of the following arguments:

### The Comparative Fallacy Is Routinely Misunderstood

The methodological decisions to prevent the *CF* entail much more than not including a control group of native speakers in the design of a study. We will argue that the *CF*, in fact, constitutes a significant barrier to providing meaningful analyses and explanations, and it does not support the fundamental explanatory nature of the field.

### Acquiring a Language Is an Incremental Process and Learners’ Grammars Develop Towards a Target

The developmental nature of L2 acquisition means that L2 speakers can be situated along a linguistic continuum (see a similar proposal by Polinsky and Kagan, 2007 for Heritage Speaker Grammars which represents different stages of acquisition and proficiencies). ILG are representations of specific points in the process of acquiring a second language as learners move closer to an end point. Since native controls are speakers who have a complete (or end state) grammar (see Meisel, 2011), it is legitimate to regard a NS grammar as a possible end point (target) in the L2 acquisition continuum. Thus, comparisons between the current state and the target (the end state grammar) as well as the current state and (a possible) next state (i.e., NNS-NNS comparisons) are necessary in order to understand the fundamental nature of ILG and L2 acquisition. Without such comparison, the data can be described but both meaningful analyses and predictions for subsequent development are virtually impossible.

### Native Controls are Necessary in Experimental SLA to Validate the Tasks

Evidence from the behaviour of native controls is key as it ensures that the instruments are appropriate and that the theoretical assumptions are correct. We advocate for the elicitation of data from a variety of tasks so any conclusions on the nature of ILG are based on more than one source of evidence. Native speakers are not chosen as the baseline because they are perfect, privileged or infallible but because they are often the control group that is methodologically appropriate. This is why native speakers undergoing attrition are not appropriate controls for SLA studies (their grammars do not represent the end state of language acquisition anymore), although they may be appropriate controls in other contexts.

### The Control Group Needs to Be Appropriate for Each Specific Study

The control group and the experimental group need to be matched on a number of variables to ensure that they differ only with respect to the condition to be investigated. Since some variation in the behaviour of NS is expected, it is essential that both groups speak the same variety (i.e., be exposed to the same evidence available in the input) and have the same level of literacy (same educational background) and same background characteristics (see Dąbrowska, 2010; Hulstijn, 2011; Andringa et al., 2012; Hulstijn and Andringa, 2014). The challenge is to decide what group of NS to include for the comparison with NNS to be appropriate.

Debates on the usefulness of native controls go beyond the legitimacy of the *CF* as many believe that comparisons between native speakers and non-native speakers are *unfair* (on moral grounds) as learners/bilingual speakers are expected to conform to native norms unfairly. This is particularly critical in the case of learners of English as this language carries added connotations of colonisation, power and privilege, notions which are not the main concern of most SLA researchers. It is important to note that the original formulation of the *CF* discusses comparisons between grammars (interlanguage systems) without specifically referring to the speakers of the target language (TL) as native speakers. However, one main objection clearly concerns the use of native speakers. There are various reasons for this, one of them being that ‘native speaker’ carries negative connotations outside the strict SLA remit. In particular, concepts such as NS and NNS are used to represent the people themselves, even though for linguistic-cognitive SLA approaches (as well as for Bley-Vroman), the focus is on the linguistic system, not the speakers in their social context.

One consequence of the misunderstanding of what the object of study is (grammars vs. people) is that any analysis or evaluation in deficit/error terms can erroneously be extended to the speaker as a person. In turn, this can be used to claim that SLA researchers think of learners as being deficient speakers (Ortega, 2014; The Douglas Fir Group, 2016). Although issues around native prestige have been debated in related fields for some time [see, e.g., discussions on the superior native speaker (Phillipson, 1992)] and native-speakerism in English Language Teaching (Holliday, 2006, 2015), these are now emerging within our field. The extreme position goes as far as arguing that grammars (or ILG) are not a legitimate object of study (see Ortega, 2019), a claim which is neither in the spirit of the original formulation of the *CF* nor does it fit within the main goals of our field.

Although we argue against using the *CF* to make methodological decisions in our research, we also recognise that those working on formal/cognitive SLA approaches should pay attention to the terminology employed and the rationale for including comparisons with native speakers when this is the appropriate choice.^3^ For instance, referring to ‘NS-NNs comparisons’ may no longer be completely appropriate in certain contexts as this is likely to be interpreted to mean that it is the speakers themselves who are being compared. It has to be clear that we are talking about systems, grammars, interlanguages and abstract grammatical representations. For that reason, we propose that in certain contexts, ‘end state grammars’ instead of ‘native speaker’ can be useful to avoid this type of confusion.

Being mindful of how we make our research findings available to non-specialist audiences is also important (see discussion in Domínguez et al., 2019), in particular when discussing notions, such as ‘deficits’, ‘incomplete acquisition’ and ‘not target-like’, which can be easily misconstrued. Criticisms based on the *CF* and the monolingual bias have increased at a time when SLA researchers working on theoretical issues are urged to share their research findings with people who are not familiar with our goals and methods, including researchers in other disciplines, the general public, funding agencies and the learners/bilingual speakers themselves. We recognise the difficulty in explaining notions such as deficits and errors, incompleteness and underproduction, but rather than rejecting the use of NS we ask to engage in a debate on how the field can overcome this challenge.

## Describing, Analysing and Explaining L2 Grammars

When the *CF* was proposed in 1983, the field of SLA looked quite different to what it does today, both in terms of its goals and methodology. At the time, there was an interest in investigating the systematicity of interlanguage grammars (ILG), the oral language produced by L2 speakers (Nemser, 1971; Selinker, 1972).^4^ One of the main assumptions of interlanguage studies, inspired by generative studies (Sorace, 1996), is that ILG are systems governed by rules. This implies that ILG are systematic, although some variation in the behaviour of learners is expected as well (see Andersen, 1977; Hyltenstam, 1977; Dittmar and Klein, 1979; Tarone, 1979; Meisel et al., 1981; Clahsen et al., 1983; Ellis, 1985; Schachter, 1986).^5^

Brey Vroman’s (BV) rationale for proposing the *CF* was based on his criticism against how systematicity was being investigated at that time by studies using oral production data as evidence. BV’s focus is on how researchers can best describe the ILG without involving the target language. In interlanguage studies, grammars are systematic if they conform to certain rules and expectations which need to be established by the researcher and are based on analyses of the target language (TL). How can the researcher know what the learner is thinking or what the ‘internal logic’ of the ILG is? For BV, this question cannot be answered with the analytical tools employed at that time, mainly searching for contexts in which a specific form *should* be used (the so-called obligatory context). Pica (1983,p. 70) explains that ‘*Suppliance in Obligatory Contexts (hereafter also SOC) is used to determine accurate suppliance of morphemes in linguistic environments in which these morphemes are required in standard English*’. The notion of SOC has been instrumental in morpheme studies (Brown, 1973; Dulay and Burt, 1974) which have focused on tracking the emergence and use of morphological forms in English. SOC was criticised at that time because it cannot reveal whether the learners have acquired all patterns and distributions of use of the target forms^6^. As Pica (1983) agues, target-like use analysis (TUA) can provide this insight as it also includes the number of non-obligatory contexts in which the target form is supplied inappropriately. A review of these two analyses by Pica (1983), however, shows that when applied to the same data set, they render different results so different interpretations can be made depending on how the target forms are quantified. The point that BV is trying to make, however, is that SOC is not learner-based as it does not emerge from observations of the learner data alone but by comparisons with the target grammar. In particular, he criticises the methodological approach described in Tarone et al. (1976) as it is based on ‘*the mistake of studying the systematic character of one language by comparing it to another*’ as well as the fact that ‘*it obscures the internal structure of the learner’s system (p.6)’*. This is the Comparative Fallacy (*CF*).^7^

BV points out a number of problems with the methodology that Tarone et al. (1976) used to investigate systematicity, including that they cannot discern subcases of obligatory context and that the applicability of their measure is unknowable because one cannot tell whether the learner is faced with a binary choice as they assume. He argues that the binary nature used in SOC studies lumps together many possible options which the learners may have entertained but which cannot be revealed by the limited nature of the options made available by the researcher. He also notes that the linguistic analysis that the researcher brings to the ILG may not be available to the learners. This implies that the mere speculation of an obligatory context is a case of a comparative fallacy. Since the description of the ILG has to be done independent of the TL, the use of obligatory context (or any assumption that a certain form should be used) is, indeed, discouraged as well. If there is no possibility of any expectation of use of a form, then other key notions, such as accuracy or errors, should not be used either. This, in turn, implies that even describing whether learners use a form using percentages (e.g., reporting that a certain form is used an x number of times), which is common practice in the field, has to be abandoned too. The point is that adopting the *CF* has a knock-on effect on the whole range of methodological choices and types of analyses available to researchers well beyond NS-NSS comparisons.

Other concepts and tools that should not be used for the same logic are proficiency scales (beginners, intermediate, advanced, near-native etc), omissions, overproductions, simplifications and all of the other typical characterisations of interlanguage grammars [for an example of an analysis without these concepts see Klein’s (1998) description of the Basic Variety]. In fact, even investigating whether a form or structure has been acquired is a case of the *CF* as this question already imposes a view of the learner system based on what is observed on the TL and not their internal logic. It is clear that adopting BV’s own interpretation that ILGs are systems in their own right is at odds with one of the main assumptions in our field: that L2 speakers are learners engaged in the process of learning the grammar of a second language and that in this process they entertain different linguistic systems until they reach the end state (the target grammar).

For this reason, it is important to understand that adopting the *CF* has important methodological consequences involving the tools that researchers can or cannot use in their research. It is often the case that researchers who choose not to use comparisons with native controls still analyse the learner data in terms of accuracy and expected use/acceptance of forms, even those this necessarily assumes the existence of a baseline and, thus, promotes the *CF*. For instance, Schwartz (1997) agrees that UG-oriented SLA suffers from the comparative fallacy because the ILG is judged against norms from the target language. However, she also claims that ‘*From this perspective, that properties of the TL do not get acquired requires explanation’*. Implicitly, Schwartz still assumes that L2 acquisition involves acquiring features present in the grammar of another group of speakers who are not the learners (i.e., absence of a required feature is an error). Schwartz’s (1997) view, with which we agree, still constitutes a case of the comparative fallacy according to Bley-Vroman’s own definition.

Furthermore, by focusing on descriptions of ILG only, BV avoids the fact that his proposed methodology makes it virtually impossible to provide meaningful explanations about the nature of ILG and the process of acquiring a second language. Thus, the main problem arising from adopting the *CF* is that it does not fulfil the explanatory goal of the field. At the time when BV proposed the *CF*, the focus may have been on providing descriptions of ILG but this does not meet the main goals of the field^8^ today which include to (1) describe (2) analyse and (3) explain the process of acquiring grammatical systems (see Gass, 1998; Norris and Ortega, 2003). Adopting the *CF* in its strong form is problematic as researchers could only (1) describe ILG but not (2) analyse or (3) explain (evaluate on theoretical grounds) the evidence. This position strongly contradicts the main goals of the discipline as stated above. A soft version of the comparative fallacy is also possible: the only goal of SLA is to (1) describe and (2) analyse ILG avoiding (3) to explain (i.e., evaluate on theoretical grounds) the evidence. This version also explicitly excludes comparisons with controls, and it is in line with the original spirit of the *CF* (i.e., to provide the right kind of descriptions emerging from the learner data only). However, it is also in contradiction with the main goal of our field as it necessarily precludes an interpretation and evaluation of any finding. For instance, if a group of learners are found to use the definite article in some contexts (a description of the data without quantifying the use by means of an obligatory context), we would not be able to interpret this finding to be low or high if we do not know what the expected use is as set by speakers who already have that form in their grammatical systems. The only way that research can provide meaningful and appropriate analyses of ILG and test hypotheses which investigate the acquisition process is by comparing ILG with the target grammar.

The Comparative Logic is the only position that can achieve the three goals of SLA research: (1) describe (2) analyse and (3) explain ILG. This position justifies the use of controls and comparisons between grammars from learners and a baseline on purely scientific grounds. The baseline for L2 studies is often formed by native speakers but this is not necessarily always the case (e.g., two groups of learners to investigate L3 acquisition; comparing second- vs. third-generation bilingual heritage speakers; comparing the native language of monolingual and bilingual speakers undergoing attrition). As we will argue in the following sections, native speakers become legitimate members of a control group because of the nature of their grammatical systems, not because they are ideal or infallible speakers. It is also possible that certain grammatical areas may be subjected to a higher level of variation than others even for native speakers. This is why it is informative to collect these data from a control group in experimental SLA studies.

In summary, in this section, we have shown two main problems with adopting the *CF*; first, researchers lose the main methodological tools and concepts which are necessary to analyse and explain learner grammars (error, accuracy, overproduction, etc); second, the possibility of providing meaningful explanations is virtually impossible if there is no link between the learner and the target grammars. We have argued that comparisons, including NS-NNS comparisons, are necessary to meet the main SLA goals, a position which we have called the Comparative Logic.

## Development in SLA: Accepting That L2 Speakers are Learners

The view that ILG are systems in their own right, which can be traced back to at least Selinker’s (1972) original definition of interlanguage, is widely adopted in our field. Bley-Vroman agrees with this view as well but also argues that ILGs need to be analysed independently of any other system as this is the only way that the own logic of ILG can be revealed; for this reason, he claims that the comparison with the grammar of speakers of the target language (TL) makes ILGs degenerate versions of the native grammar. It is important to note that the word chosen by BV is ‘degenerate’ which means degraded, abnormal and of lower quality. In our view, degenerate is an unfortunate choice of term as it is a measure of quality (i.e., non-native speakers produce language of substandard quality) which does not naturally arise from the objective description of that system.

Some researchers have taken the view that if L2 grammar lacks a grammatical feature or contains an error, that means that the speakers themselves are deficient in some way (see Firth and Wagner, 1997).^9^ Although this misconception has been already addressed by some (see Gass, 1998), criticisms of this kind towards linguistic-cognitive SLA research still remain (Ortega, 2014, 2019). Reconciliation on this matter necessarily entails an understanding of how ‘deficit’ is understood in linguistic-cognitive SLA and why it is important that we investigate both what learners can and cannot do in the process of acquiring a second language. Although a deficit view of acquisition (both for first and second-language acquisition) exists, this is to mean that learners make errors or show incomplete knowledge of a certain grammatical aspect of the TL, not that the learners themselves are deficient in any way. Both fossilisation (Selinker, 1972; Han, 2004) and incompleteness (Schachter, 1988, 1990; Sorace, 1993) have been routinely used to describe aspects of learner grammars. These terms only make sense because ILGs are evaluated against a target (complete) grammar where target means that it represents the outcome of language acquisition under ideal input conditions (what we will characterise in the next section as the ‘end state’). We have already argued that since the *CF* prevents researchers from making any evaluations of ILG that would conclude that the system is degenerate (incomplete or deficient), concepts which are widely used in our field, such as errors, omissions, overgeneralisations and simplifications, would need to be abandoned as well. In our view, this is the wrong approach as we would stop using the tools that allow researchers to carry out explanatory research in second-language acquisition. For this reason, it is our view that any research committed to offer precise descriptions and explanatory answers will necessarily be subject to, at least, the soft version of the comparative fallacy as any explanation arising from the description of ILG would necessarily need to address the deficit/error issue we just noted.^10^

In fact, adopting the idea that the *CF* exists intrinsically threats the notion of interlanguage itself, as interlanguage was proposed as a means to account for the process involved (often shown by different stages) in learning a target language. In traditional interlanguage studies, assuming that learners develop a second language (i.e., they move closer to the TL) does not necessarily mean that an ILG is not a system in its own right but, rather, that the learner is in the process of acquiring a full grammatical system with all the features expected in that system. For instance, Spanish has grammatical gender which triggers a type of agreement between nouns, adjectives and determiners (e.g., *la gata negra*/the black female cat). Thus, it is reasonable to expect that learners of Spanish will have to learn this feature which is likely not to be present at the early stages of acquisition. Until that feature is present in their grammar, the process of acquiring Spanish (the target grammar) can be said to be incomplete. Researchers interested in finding out how learners go about the challenge of acquiring a new feature (gender) which does not exist in their native language need to know whether learners use the right gender (masculine or feminine) appropriately. It would not be possible to do this without a reference to how gender is used by speakers of the target language.

In this respect, one basic assumption in SLA studies is that we are investigating a process whereby a speaker *develops* a second/n language through a specific route. An ILG represents specific points in the process of acquiring a second language (see Meisel et al., 1981 for a discussion on developmental stages in L2 acquisition). This process necessarily entails a progression which, in turn, necessarily assumes that certain features of the target grammar can/should/will be absent. In this respect, one can argue that this represents a ‘deficit’ view of language development, similar to what is observed in the process of acquiring a native grammar in the case of children, as a learner starts the process of acquiring a language with very little knowledge of the grammar which is being learnt. Deficit in this context does not mean that the grammar of a learner is of lower quality, degraded or degenerate, nor is it an evaluation of the speakers themselves. It means that the system entertained by the learner does not (yet) show the features and properties of the target grammar. Importantly, this ‘deficient’ or ‘incomplete’ view is not in opposition to the view that learner grammars are systems in their own right.^11^ Both interpretations can be true. This point becomes clear when analysing overregularisations, such as when learners use the English past tense marker *-ed* with an irregular verb (e.g., using ‘goed’ instead of ‘went’). The use of ‘goed’ is both an error (i.e., it is not how the verb ‘go’ marks past tense in English) and the result of the speakers’ grammar respecting a certain grammatical principle of their own system (e.g., use ‘ed’ with all verbs to mark past tense).

We would like to reiterate our point that without comparisons with the TL, there can be no analysis, and that without analysis, there cannot be any explanations. Notions, such as accuracy and errors, are fundamental to understand the processing of acquiring a language in all contexts. There are numerous examples of how different SLA frameworks make notions, such as accuracy and error central to their analyses. Without these, there would be no field. A good overview of some ways in which interactionist, emergentist and generative scholars measure SLA is found in Norris and Ortega (2003). For instance, these authors show that detecting the use of a form is important for interactionist approaches to SLA. However, this is not the measure use for acquisition as learners have to show that they are also able to use that form *appropriately* and fluently. The only way in which it makes sense to describe the use of a form as appropriate is if some criteria for such use has been established.

With regard to emergentist approaches, Norris and Ortega (2003, p. 727–728) explain that accuracy is one of the main factors used for establishing the parameters of acquisition in this framework. As in the case of appropriateness above, accuracy can only be established if a comparison with a ‘correct’ use of the form is established. In these two frameworks, comparisons between learner grammars and the grammar of the TL are necessary to fulfil our goal of understanding the process underlying SLA. Interestingly, the term ‘nativelike’ is only mentioned by Norris and Ortega when they describe generative approaches to SLA: ‘*Generative linguistic studies of SLA are likely to rely almost exclusively on the outcomes of grammaticality judgment tasks of various kinds, where acquired means nativelike levels of rejection of illegal exemplars of the target grammar’*. Although we agree with these authors that the term nativelike is often used by generative SLA studies, the same concern with the appropriate and accurate use of target form is shared by all of the frameworks reviewed by these authors. For all these researchers, the use of target forms is analysed by comparison with a group of speakers which perform target-like. That is, one fundamental notion of acquisition is that it assumes conformity with native use/judgement in all approaches. For instance, in a study promoting task-based learning, Pica et al. (2006, p. 320) describe ILG as being full of omissions, substitutions and inconsistencies and a varying degree of accuracy. They do this without explicitly comparing learner behaviour with a group of native control even though this is the only one in which they can discuss accuracy. An important body of research has been concerned with the role of corrective feedback in SLA. Studies on corrective feedback assume that L2 learners make errors. For instance, Ellis et al. (2006, p. 340) argue that ‘*Corrective feedback takes the form of responses to learner utterances that contain an error. The responses can consist of (a) an indication that an error has been committed, (b) provision of the correct target language form, or (c) metalinguistic information about the nature of the error, or any combination of these’*. Superficially, one could conclude that the focus of the investigation is to show that learners fail to acquire a second language. Similarly, approaches which investigate NS and NNS interactions (see, e.g., Lyster and Saito, 2010) do so on the assumption that the NS plays a crucial role in second-language development: it is through the interaction with a NS that input is rendered comprehensible to learners. Finally, when Andersen and Shirai (1994, p. 143) proposed the extremely influential ‘Aspect Hypothesis’ to explain the L2 acquisition of past tense morphology, they were trying to explain why learners fail to supply past marking in obligatory context much more frequently with some predicates than with others. The analysis of correct and incorrect compliance of target forms was the basis of Andersen and Shirai’s analysis later adopted by a large number of studies.

These examples show how in all of these approaches, notions such as accuracy, progress and errors, are crucial if it is expected that ILG develops towards a target. As Lardiere (2003) argues, even those approaches/researchers who are supposed to be respectful of the comparative fallacy (because they claim that they investigate learners’ interlanguage on its own right) are susceptible of it once they base their analysis on notions, such as obligatory context, accuracy and omissions. In our view, understanding and explaining SLA necessarily require comparisons with a baseline. We have called this the Comparative Logic and have argued that it is the most appropriate position in order to both view ILG as system in their own right and provide meaningful explanations. Analysing and understanding when success is both possible and when it is fundamental in our field.

## Generative SLA and the Role of Native Controls

In the previous section we showed that comparisons between learner and complete (native) grammars are commonplace in the field because they are necessary to explain ILG irrespective of the theoretical framework; however, it is often the case that researches in the generative tradition are the target of criticism specifically for promoting comparisons between learner and native grammars and for the (erroneous) belief that the field sees native speaker norms as a goal. This is partly due to the fact that having evidence from native speakers’ intuitions is clearly part of the methodological design. There are other reasons which are linked to the main assumptions of the whole generative enterprise which have been carried over to SLA research. As we have already explained, generative SLA is concerned with the abstract linguistic knowledge of speakers, what they unconsciously know about language(s). The field assumes an innate and biologically determined capacity for language which is unique to humans. The specialised and abstract module specific to language known as the computational system includes a lexicon and the syntactic operation Merge (Chomsky, 1998; Berwick and Chomsky, 2008; Friederici, 2017) which builds syntactic structures which are interpreted and pronounced by specific subsystems. Importantly, there is evidence suggesting that access to this capacity may decline with age as differences between how speakers acquire a native and a non-native language have been found (see discussion in White and Genesee, 1996).

Unlike other cognitive approaches, generative SLA is interested in I-language, rather than language as a social or cultural object. I-language is an internalised system, what is also known as a grammar. I-language is according to Chomsky et al. (2019) ‘*a system that links meaning and sound/sign in a systematic fashion, equipping the speaker with knowledge of these correlations’*. During the language acquisition process, assumed to be constrained by Universal Grammar (UG), children develop a grammar (i.e., they figure out what is correct and what is not) and establish form and meaning pairs as determined by the language faculty (Chomsky, 1986). These form-meaning connections thus exist in the target language which serves as the input for L2 speakers. Typically, the language acquisition process finishes when children’s grammars reach the so-called ‘steady’ or ‘end state’. The ‘steady state’ is the full adult grammar resulting from full access to UG and exposure to a full set of linguistic input; in this respect, one could say that it is what results in ‘ideal conditions’ for language acquisition in the sense that full convergence with the ‘end state’ is always achieved. For this reason, we argue that a more appropriate way of calling native speakers in SLA research would be ‘end state speakers’ or even more appropriate those who have an ‘end state grammar’ to avoid any confusion about what the object of our study is.

In the context of L2 speakers, ILG is also an I-language (see Adjemian, 1976; Klein, 1998). L2 speakers have access to UG^12^ during the acquisition process but the characteristics of their ‘steady state’, unlike the case of children, are unclear. It is also not completely obvious whether any intermediate grammars or ILG have direct access to UG or whether all L2 speakers reach a similar ‘steady state’ with the same characteristics.^13^ Comparing the status and characteristics of these intermediate I-languages and the corresponding ‘end states’ is useful to evaluate the role and accessibility of UG, the role of the input during acquisition, L1 influence, etc. Even though updated views of the role of UG have promoted other types of research questions (the role of linguistic interfaces, representational impairment vs. computational efficiency, feature-reassembly etc.), White’s (2003) claim that ‘*the crucial question is whether or not interlanguage grammars are UG-constrained, rather than whether or not they are native-like*’ is still valid today.

One specific and very common criticism against generative SLA is based on the (misinformed) claim that generative approaches to language are based on native speakers are idealised speakers (Leung et al., 1997); embedded in this criticism is, again, that speaker here refers to the speaker as a person functioning in the real world, not their abstract linguistic system as we have just explained. This particular criticism often arises from a misunderstanding of what ‘ideal’ means^14^ and the reasons that led Chomsky to propose this assumption in the first place. The contentious quote from Chomsky (1965, p. 3) is as follows: ‘*Linguistic theory is concerned primarily with an ideal speaker-listener, in a completely homogeneous speech-community, who knows its language perfectly and is unaffected by such grammatically irrelevant conditions as memory limitations, distractions, shifts of attention and interest, and errors (random or characteristic) in applying his knowledge of the language in actual performance’*. This may appear to be a call for a search of the perfect speaker, which is identified with a native speaker (i.e., nativism equals perfection). However, Chomsky is really arguing that in order to understand grammar as a cognitive system (competence), one has to look further than what speakers actually say (performance) as this is modulated by non-linguistic factors. Chomsky is concerned with knowledge of a grammar as an abstraction, an outcome of language acquisition in ideal learning conditions, not as a real object that can be studied. In this respect, Chomsky (1965) also explains that ‘*a generative grammar is not a model for a speaker or a hearer. It attempts to characterize in the most neutral possible terms the knowledge of the language that provides the basis for actual use of language by a speaker hearer’*.^15^ Critics also ignore the fact that Chomsky later abandoned the competence-performance distinction (and the idealised speaker) in favour of I-language and E-language (Chomsky, 1986) as this distinction, among other reasons, can account for linguistic variation.

There have been some attempts to deconstruct and even rethink the need to assume an ideal speaker/hearer both in formal and experimental contexts. For instance, Chesi and Moro (2015) discuss the competence vs. performance distinction proposing that there is both an idealised native speaker and a real native speaker. The native speakers are not idealised speakers themselves but have access to the grammar which is the object of study. This distinction is also useful for explaining why the behaviour of native controls does not always agree with the predictions made by linguistic theory (which are based on the most idealised competence systems). Similarly, in the SLA literature, Duffield (2003) distinguishes between two types of linguistic competence (underlying and surface competence) to account for knowledge of gradient grammaticality (when a structure is more acceptable than another). Underlying competence is categorical, whereas surface competence is more probabilistic as it includes several factors, such as sensitivity to frequency of constructions. More recently, Slabakova et al. (2011) have provided empirical support for Duffield’s dual competence system and Sorace and Keller (2005) have also made a similar distinction between hard (syntactic) and soft (interface-based) constraints which yield different levels of acceptability.^16^

The use of native speakers as native controls has been often justified on methodological grounds. Research which focuses on judgement data as the main source of evidence requires a control group in the experimental design. Since the control group is very often a group of native speakers of the TL, although not exclusively, the comparison between native and non-native behaviour is often made explicitly. As Sorace (1996, p. 380) notes: ‘*For the correctness of judgments to be empirically assessable, it should be possible to measure intuitions of degree of grammaticality against some independently established grammaticality scale’*. Sorace’s quote shows that comparisons among groups (including a baseline of native speakers) are necessary for explaining and assessing the results arising from linguistic judgements. It is an essential part of the experimental design used in research which investigates learners’ judgements and intuitions. In this type of experiments, a set of variables are defined and controlled. The control-experimental group comparison is also necessary to determine whether the results are the effect of the independent variables or not, to establish the baseline of comparison, to verify the validity of the task and for investigating whether the hypotheses are incorrect and need to be reformulated. There are numerous examples showing that this has been the case in Generative SLA. For instance, Grüter (2006) in a key study which found support for the Full Transfer/Full Access position used a control group to analyse the acquisition of wh-questions in German. The behaviour of the control group is key to show that there is a bias for one of the two possible readings of a question which was not expected nor found in the L2 data. Without the native control data, some of the learner behaviour would have not been explained by the hypotheses.

If native speakers are necessary as baselines to control conditions and offer a key measure for understanding learner behaviour, how can researchers meet their methodological needs and avoid the *CF* at the same time?^17^ This is definitely a challenge for UG-based research which has an explanatory goal that goes beyond providing descriptions and often elicits intuitions; in fact, such is the difficulty that we argue that it is virtually impossible. In our view, the key is to separate that comparisons between native and non-native grammars are necessary from any conclusions that researchers can reach based on those comparisons (the issue is how ‘deficit’ is/should be approached). In particular, it should be possible to investigate learner grammars in their own right while providing analyses which take into account the judgements of speakers of the target language. In this respect, we agree with Sorace (1996, p. 385) that even when the comparison with native speakers are justified ‘*learners’ judgments themselves should provide the primary criterion for deciding which structures are or are not part of it (the non-native grammar)’*. The learner’s data are still the relevant data as argued by Birdsong (1989).

Those researchers which still choose not to include a group of native speakers as control groups need to clearly specify how they intend to provide accurate and appropriate descriptions and explanations of the learner data. For instance, Heil and López (2020) included a group of native speakers as controls but learners’ and natives’ judgements were not analysed together. The authors showed the results of the monolingual English group in order to verifying experimental validity as they wanted to avoid the *CF*. In the method they use, they provide evaluations of learners’ grammars based on indirect comparisons with native controls. However, it is difficult not to draw comparisons between these two groups when both sets of results are presented together in the same tables and there is a clear connection between the behaviour of the learners and the native speakers. Furthermore, there are studies in which a direct statistical comparison between the control and the experimental groups is justified. This comparison, which is essential in certain studies, should not be ruled out on the basis that it provides a case of the *CF*.

## Variability in the (Native) Control Data

One final argument against NS-NNs comparisons is that the NS themselves do not form a homogeneous group and variability in the data makes it difficult to set goals for learners based on how we expect NS to behave. In this section, we argue that variation within a community of speakers and within speakers themselves is nothing unusual and has been successfully accounted for in linguistic theory. We will also show how some of the concerns raised with respect of variability can be mitigated by applying more rigorous research methods, in particular better sampling techniques.

Formal SLA has borrowed analytical tools from linguistic theory as researchers assume that evidence of knowledge of grammar is shown by knowing what is both grammatical and ungrammatical. There is also a long tradition of testing hypotheses in controlled, experimental settings.^18^ *A priori* it may seem that variation is problematic for a UG approach to language since UG is invariant by nature. However, variability has been accounted for by several approaches, such as Adger’s (2006) Combinatorial Variability model or the Multiple grammars approach (Kroch, 1989, 1994; Yang, 2002). Another recent development has brought together generative syntax and variationist sociolinguistics [see review in Adger et al. (submitted)] and employs a new methodological approach which moves beyond the individual and focuses on both linguistic and social aspects of the whole community of speakers. Under this approach and following Labov (1982), it is expected that the linguistic rules shared in the community are of a variable nature. Sentences which would be ungrammatical for some speakers of English can be part of the grammar of speakers of certain varieties for which the standard and the regional variety are both possible. For instance, Henry (1996) shows cases of word order variation with imperatives in Northern Irish English as shown in (1a) and (1b):

1.

- You go away
- Go you away

Despite the fact that intra-speaker variation is often observed as shown in example (1), there is still an expectation that speakers would conform to certain rules, that is certain aspects of the grammar are not subjected to intra-speaker variation regardless of differences in gender, class, style, education, age etc. For instance, sentence (2b) with a missing subject would not be acceptable by any speaker of English:

2.

- Lena says that [she] will come soon
*Lena says that [] will come soon

There is some tension between conformity and variability when investigating the linguistic behaviour of speakers. We expect speakers of English to conform to core syntactic properties (such as the use of overt/null subjects) in some cases more clearly than others. It is important to highlight that cases, such as the examples shown in (1) are cases of *true* variability in the speakers’ grammars (I-language). However, the SLA literature also describes a type of variability which is linked to performance and to other methodologically related issues. For instance, Sorace (1996, p. 377–378) mentions several extralinguistic factors that are likely to influence how participants go about completing grammaticality judgements including parsing strategies, context and mode of presentation, pragmatic considerations, mental states and linguistic training. Schütze (1996/2016) also shows that literacy is a relevant factor. These and other similar factors, which are external to the mental representation of the grammar, are important for SLA researchers and can affect the results arising from grammaticality/acceptability tasks giving raise to extralinguistic variation. Researchers should try to minimise this by choosing the appropriate design and research method.

In particular, it is important that for some structures, researchers allow for the possibility of using gradience or a range of responses (usually a Likert scale) rather than restricting the responses to yes/no answers (see discussion in Schütze, 1996/2016). In some cases, it may be necessary to elicit evidence through various types of tasks and make comparisons based on a range of answers rather than a fixed point (see, e.g., Hyltenstam and Abrahamsson, 2000; Abrahamsson and Hyltenstam, 2009) and how they judged the performance of L2 speakers against the whole range of responses provided by native speakers). Recruiting participants to be part of the control group is an important task which needs careful attention from the part of the researcher (see Lipsey, 1990; Quené, 2010) so that the sample is both as homogeneous and representative as possible. Special attention needs to be paid so that the control group and the experimental group are matched on the key variables to ensure that they differ in respect of the condition to be investigated only. Other adjustments, such as that both groups speak the same variety and are exposed to the same evidence available in the input, should be taken into account as well.

It has also been argued that other factors, such as processing and experience, may be subjected to variation. When investigating individual differences in L2 acquisition, Andringa et al. (2012) assume variation in listening proficiency for both non-native and native speakers. They found that the success comprehension process for native speakers depends on their ability to deal with the pressure of online speech processing. Those speakers with more accumulated experience processing complex texts were the best listeners. This suggest that NS should be matched with NNS of similar literacy levels. A similar argument has been made by Hulstijn and Andringa (2014) as they argue that it may not be possible to single out a single factor responsible for variation in their native control data as effects of working-memory capacity, reasoning ability and reaction-speed in a nonverbal task together could explain effects of age and length of exposure. In general, these and other studies investigating individual differences reach the conclusion that NS-NNS are legitimate as long as the right NS are included in terms of literacy, educational background, experience, background characteristics etc. Individual variation can also be an effect of the task. In this respect, Hulstijn (2011, p. 236) shows how individual differences in some tasks employed are mainly restricted to differences in the speed with which linguistic information can be processed (as a function of age), whereas in other tasks, it is ‘mainly by differences in intellectual skills and amount of reading and writing activities, as reflected by education, occupation and leisure-time activities’.

Finally, the type of predictions and expected results can have an effect on the results as well. For instance, in our investigation of the use of preterite and imperfect forms in Spanish by both native and non-native speakers, we asked all the participants to complete a series of oral and comprehension tasks (see Domínguez et al., 2013). We investigated whether the predictions of Andersen and Shirai’s (1994) Aspect Hypothesis (AH) hold for both groups so it was important to have data showing the use and acceptability of the target forms for the native speakers and the learners. According to the AH, preterite tends to be used with telic events rather than with atelic events; on the other hand, the imperfect is preferred with atelic events. The results of two oral production tasks, an interview with an investigator and a picture-based story retell show that, despite some variation in the amount of preterite and imperfect forms produced by the controls, the averages conform to the expected results. For instance, in the interview, the least-controlled task, the native controls used the preterite with achievement (telic) verbs on average 80% of the time, whereas they use this form on average 32% of the time with state (atelic) verbs. Although most native speakers used the preterit between 80 and 95% of the time with achievements, the range of use was wide from 57 to 100%. The range of use of the preterite with states was equally wide from the lowest use of 7% to the highest use of 55%. Despite this variation, the means were useful as they corroborated our predictions and showed differences with the pattern of use shown by the learners. We were able to conclude that the pattern of use of preterit and imperfect predicted by the AH is already represented in the pattern of use of these forms in the native input, so learners have access to that kind of evidence though the course of acquisition.

In Domínguez and Arche (2014), we reported variability in the data of the (native) control group even though this was not expected. All the participants completed a content-matching acceptability task to investigate preference of SV and *VS* orders with different types of verbs (accusative and unergative) and different types of pragmatic contexts (narrow focus on the subject or not). The theoretical analysis adopted predicted that native controls would prefer the *VS* structure with narrowly focused subject with unergative verbs (smoke, dance, sneeze and cry). However, the aggregated means of all the native participants showed that these speakers only chose this structure 45% of the time. A closer look at the individual results revealed that this was not a case of optionality, as native speakers had clear patterns of behaviour as roughly half of them preferred SV and roughly other half preferred *VS* in this context. Interestingly, the advanced learner group also showed variability in their responses, but in this case, the same participant would choose both options. Unlike the native controls, learners did show optionality in their responses. Based on the responses of the native control data, we were able to suggest that the input can be vague with respect to SV and *VS* structures in Spanish which can lead to difficulties (optionality) for learners.

In this section, we have argued that variation in the data is not unexpected and can be accounted for both theoretically and empirically. A more careful selection process for the control group can mitigate problems arising from extralinguistic variation and ensure that the sample is representative and appropriate.

## Will These Issues Ever be Resolved? Some Reflections for the Future

A review by Zuengler and Cole (2005) shows that criticisms against the goal and methodology employed by cognitive approaches to SLA have been raised for quite some time. In that review, it was clear that the criticism came from scholars from the socio-cultural tradition (e.g., Firth and Wagner, 1997). It is now the case, however, that questions on the role of the native speaker are being asked from within the cognitive field. We have analysed Bley-Vroman’s Comparative Fallacy and examined the validity of its assumptions in the context of SLA research today. We have concluded that by ignoring the target grammar, the *CF* does not enable researchers to achieve the main goals of our field. This is because making methodological choices on the basis of the *CF* entails much more than not including control groups of native speakers. Those who choose to avoid the *CF* would not be able to make any *a priori* predictions that would impose their own analysis/expectations on the learner data; they would not be able to analyse the data in terms of what is not produced, whether forms are absent or overused or simplified etc. The analysis they produce will not be able to make references to errors or accuracy either. Since these are notions which are essential to account for the nature of the acquisition process, we conclude that adopting the *CF* will prevent researchers from providing meaningful explanations.

We have also argued that the only position which ensures that the goals of the field are met (describing, analysing and explaining the process of learning a second language) is the Comparative Logic, the view that comparisons with a control group or baseline are necessary. The field, almost 30years after BV proposed the *CF*, is well-equipped to make comparisons between learner and non-learner grammars in a way that respects the principle that ILGs are systems in their own right. Nevertheless, careful attention needs to be paid to the methodology chosen and, in particular, the sampling process for inclusion of participants in the control group or the baseline for comparison. Researchers should consider not just what types of tasks to employ but also how variation in the cognitive skills, literacy, experience etc. of the participants in the control group could lead to variability in the results.

We have also argued, as others have before us, that for cognitive SLA, errors are an important source of information when investigating the learners’ mental grammars. SLA is a process by which learners entertain different interlanguages or I-languages which may not include all of the features of the target grammar until they reach the ‘end’ or ‘steady state’. The ‘steady state’ is the adult grammar which results from the interaction of UG, exposure to input and certain cognitive principles during child language acquisition. Interlanguage is a type of I-language, an abstract, subconscious and internalised grammar with characteristics similar to learner grammars (ILG). For this reason, we completely agree with Gass (1998, p. 84) when she claims that the scope of inquiry of SLA is to study acquisition and so L2 speakers in this context are necessarily *learners* and not *users* of the language.^19^ In the same spirit, we emphasised in this article that in order to answer relevant questions about the nature of ILG, we need to focus on the grammatical systems and not the speakers. Crucially, our enterprise does not preclude others from studying social aspects associated with learning a second language.

We are mystified that anyone could conclude that our field *promotes* native speaker norms and that there is a monolingual bias in SLA (see, e.g., Kachru 1994). We hope that this article has shown that there is no privileged status or prestige associated with the notion of a native speaker *per se* nor that native speakers are a model or inspiration for learners [see Davies (2003) for this view]. Criticisms of this sort are particularly common when generative SLA is targeted, as it is often criticised for focusing too much on correctness and the native norms. We have shown that this is due to a misunderstanding of our goals and scope of inquiry. Since the emphasis is of generative SLA is on understanding grammars (as opposed to communication or language use) and we directly judge learners’ intuitions as grammatical or not, some may think that the field sees correctness as a goal when this is not clearly the case. Nevertheless, we admit that there needs to be more clarity from our part on our goals and methods, particularly when sharing our research with non-experts. In this sense, a clearer rewording of our research questions would be a step forward. For instance, generative SLA does not investigate *if an L2 speaker can become a native speaker* but rather *if an end or steady-state grammar can be attained based on partial input after the onset of the critical period*. The problem we see with this is that the latter is harder to understand and it is not as attractive as the former, particularly as researchers are under pressure to get funding, make our research impactful to non-specialists and seek collaborations with other disciplines.^20^

We believe that this is a serious issue for cognitive approaches to SLA and generative approaches in particular. Although some good attempts to made formal SLA useful to foreign language teaching exist (Whong et al., 2013; Leal and Slabakova, 2019; Rankin and Whong, 2020), a large body of our research does not have an immediate application outside the academic remit, mostly because our concerns are theoretical in nature. This may be seen as a limitation compared to other approaches, when it clearly is not, nor does it justify a radical methodological change. Without research which engages with theoretical questions, there cannot be any scientifically inspired applications. Gregg (1996, p. 75) already cautioned that L2 theories may only have intellectual value since the problems tackled are fundamentally theoretical (as opposed to practical problems). Furthermore, Newmeyer (1988) also argue that ‘*progress in L2 acquisition theory, as in any other scientific discipline, comes by focusing on the explanatory problem, and not by looking over one’s shoulder at the possible applications’*. The apparent (lack of) immediate applicability issue has become quite real recently for researches working on theoretical issues. As pressure mounts to make our results meaningful and impactful in the real world, we make ourselves vulnerable as opportunities for misunderstanding multiply. Something as simple as proposing as a vision of SLA based on transdisciplinarity (The Douglas Fir Group, 2016)^21^ is likely to instigate even more criticism against cognitive and formal approaches to SLA as we are singled out for not taking into account the learners’ social context and that they are people who function in the real world. It is in the sense that transdisciplinary in the SLA context is a trap and not a vision all researchers see as beneficial for the field (see also Han, 2016).

## Data Availability Statement

The original contributions presented in the study are included in the article/supplementary material, further inquiries can be directed to the corresponding author.

## Author Contributions

LD and MA contributed to conception and design of the study. LD wrote the first draft of the manuscript. Both authors contributed to manuscript revision, read, and approved the submitted version.

## Conflict of Interest

The authors declare that the research was conducted in the absence of any commercial or financial relationships that could be construed as a potential conflict of interest.

## Publisher’s Note

All claims expressed in this article are solely those of the authors and do not necessarily represent those of their affiliated organizations, or those of the publisher, the editors and the reviewers. Any product that may be evaluated in this article, or claim that may be made by its manufacturer, is not guaranteed or endorsed by the publisher.

## Footnotes

^1^Linguistic-cognitive approaches include researchers working on cognitive-interactionist, instructed, psycholinguistically oriented, usage-based and Generative SLA according to Ortega (2014, 2019) who claims that their ‘*main disciplinary goal is to illuminate the human capacity for language, and most would also share post-positivist logics, quantitative rigor, and generalizability as values in their research* (Ortega, 2019, p. 23)’.

^2^Tensions between the so-called cognitive approaches to SLA (those which investigate linguistic systems) and sociocultural perspectives (those which focus on the socialisation aspect of language learning) are not new (see Zuengler and Cole, 2005 for a review). A good example of the types of criticism that cognitive SLA has endured over the years can be found in the arguments put forward in Firth and Wagner (1997) and the defence in Gass (1998) and Long (1997). We see recent criticisms of the type expressed in Ortega (2014, 2019) as another turn of the screw in the quest for dismissing any serious inquiry into second-language acquisition which has a theoretical interest and focuses on investigating grammatical knowledge.

^3^A type of social injustice exists in Academia as English is the dominant vehicular language for transmission and dissemination of research findings. Today, most of the high-impact journals in our field overtly or covertly support a policy by which authors must comply with English native-like writing form and style (see Costello, 2020 for the privilege of English in academic publishing).

^4^For an overview of some main interlanguage studies, see Han and Tarone (2014) and Tarone (2018).

^5^It is important to note that Selinker (1972) originally proposed that ILG can only be studied when the learner engages in oral communication, a view which was not shared by Corder (1981) who argued in favour of investigating the learner’s judgment on grammaticality as well.

^6^Methodological issues in morpheme studies have also been raised by Andersen (1977), Hakuta (1976), Hatch and Wagner-Gough (1976), Lightbown et al. (1980) and Stauble (1981).

^7^We note that although there is no explicit mention of the native language by Bley-Vroman, in principle, comparisons between ILG and any other language (native or not) would also fall foul of the *CF*. This would include comparing L2 and L3 speakers or two groups of bilingual speakers (e.g., second vs. third generation heritage speakers) or even comparing the same group of learners at different points in time in a longitudinal study. The specific objection to imposing native norms on learner grammars, although related, is in fact independent of the *CF* even though they are usually interconnected.

^8^See Zuengler and Miller (2006) for a discussion on the main opposing perspectives in SLA research focusing on the long-standing debate between cognitive and sociocultural approaches.

^9^For a similar argument in the context of heritage language acquisition, see DomÃ­nguez et al. (2019).

^10^As an example, the Basic Variety was proposed with this objection in mind and it is the result of a description of a learner system without references to a TL. This approach is rather limited in its explanatory power and the kinds of predictions it can make about the SLA process more generally.

^11^One obvious consequence arising from the developing nature of ILG is that learners are often classified according to different proficiencies. This classification assumes a comparison as well, not between learners and native speakers, but between learners and other more or less advanced learners. Since a beginner learner is only a beginner in comparison with a more proficient learner, this type of comparison should also present a case of the *CF* as per BV’s definition. This means that across-group comparisons between learners (not just native speakers) are to be avoided as well.

^12^Whether UG is available for L2 acquisition has been a major topic of debate (see Borer, 1996; Epstein et al., 1996), the current view being that learners can indeed access UG when they encounter input which cannot be comprehended/parsed by their existing grammar (the Full Access position; see White, 2003).

^13^Bley-Vroman (1990, 2009) argues that the process of acquiring a first and second (foreign) language are fundamentally different. The Fundamental Difference Hypothesis (FDH) proposes that whereas child language acquisition may be constrained by UG (which is domain-specific), foreign language learning is not, and so adults need to resort to general learning mechanisms in an instance of general skill acquisition. He makes this claim on the basis of certain observations which have mostly been contested or not completely accepted including that ‘complete success is extremely rare, or perhaps even non-existent’ (1990:6); adults not only generally do not succeed, they also fail to different degrees (1990:7); and adults set their own goals and can fail, this leads to variation in the process and outcomes. It is interesting that despite the fact that the FDH and the Comparative Fallacy are based on a clear anti UG position, some UG scholars seem to embrace both.

^14^It is only by accident that the ‘idealised’ speaker coincides with the native speaker. Some native speakers are not appropriate as control groups in L2 studies, namely, those undergoing grammatical attrition (see DomÃ­nguez, 2013).

^15^Traditionally, generative SLA research is often difficult to translate and apply to the real world (as opposed for instance to pedagogy-oriented SLA). As an example, it is not immediately obvious how examining UG accessibility can be of any use to foreign language teachers or even to the learners themselves.

^16^It is generally agreed that grammaticality taps into speaker’s competence (or I-language) and is not open to gradience whereas acceptability involve the speakers’ performance and is gradient in nature. This is because speakers are judging sentences according to their perception of those sentences (Bard et al., 1996). For a full discussion on the differences between grammaticality and acceptability plus how the parser can also affect acceptability, see Leivada and Westergaard (2020). For a detailed discussion on how the interpretation of gradient judgments affects sentence acceptability, see Francis (2021).

^17^One anonymous reviewer suggests that we consider the suitability of using native speakers who are also L2 learners as controls in SLA studies on the grounds that both groups would be bilingual. It is our view that the characteristics of the control group depend on the research question to be investigated and so studies who are interested in investigating the effects of the bilingual experience should take this variable into account when selecting the controls. In some studies having two control groups (one formed by monolingual speakers only and one formed by monolingual speakers who also know another language) may even be relevant. The reviewer’s suggestion is consistent with our view that the selection of the control group should be carefully considered for each particular study.

^18^In theoretical syntax the research method is to obtain a judgement of the acceptability of a sentence often by just using the intuition of the author/s of the study. Phillips (2009) and Adger (2014) have both defended this method. Experimental evidence supporting the validity of introspective judgements can be found in Sprouse et al. (2013) as they show that these data have medium to large effect sizes. Sprouse (2011) argues that the results of an acceptability judgement task conducted *via* Amazon.com’s Mechanical Turk (AMT) are almost indistinguishable from the results from the same task conducted in a laboratory with control from the researcher. AMT has the key advantage that it can recruit participants for the control group from a wide pool and varied backgrounds. In contrast, Gibson and Fedorenko (2013) point out some of the problems including that this method leads to incorrect generalisations due to cognitive biases from the part of the researcher. They argue for a quantificational approach using Amazon.com’s Mechanical Turk (AMT).

^19^The user-learner distinction is key to understand existing opposing approaches to English language learning and teaching: whereas SLA researchers investigate how L2 speakers go about learning a language, English as a *Lingua Franca* (ELF) researchers view these speakers as users engaged in communicative practices (see Seidlhofer, 2001; Jenkins et al., 2011). We would like to emphasise that in our view, a learner of a language can also be a user of that language in other contexts and that both approaches are not mutually exclusive.

^20^We think of this situation as a paradox. The field is urged to open up and make its research impactful to others as a way to prosper and become relevant but by doing so researchers accidentally create opportunities for misunderstanding, criticism and alienation (for instance not understanding that we are interested in analysing grammars and not people in real situations). This, in turn, raises concerns about our theoretical and methodological assumptions which can make our field less prosperous and less relevant in the end.

^21^The transdisciplinary agenda ‘*seeks to integrate the many layers of existing knowledge about the processes and outcomes of additional language learning by deriving coherent patterns and configurations of findings across domains* (The Douglas Fir Group, 2016, p. 20)’. The starting point of this agenda is the social-local reality of L2 learners.
